# The Biphasic Effect of Vitamin D on the Musculoskeletal and Cardiovascular System

**DOI:** 10.1155/2017/3206240

**Published:** 2017-08-23

**Authors:** Armin Zittermann

**Affiliations:** Clinic for Thoracic and Cardiovascular Surgery, Heart and Diabetes Center North Rhine-Westphalia, Ruhr University Bochum, Bad Oeynhausen, Germany

## Abstract

This narrative review summarizes beneficial and harmful vitamin D effects on the musculoskeletal and cardiovascular system. Special attention is paid to the dose-response relationship of vitamin D with clinical outcomes. In infants and adults, the risk of musculoskeletal diseases is highest at circulating 25-hydroxyvitamin D (25OHD) concentrations below 25 nmol/L and is low if 40–60 nmol/L are achieved. However, evidence is also accumulating that in elderly people the risk of falls and fractures increases again at circulating 25OHD levels > 100 nmol/L. Cohort studies report a progressive increase in cardiovascular disease (CVD) events at 25OHD levels < 50 nmol/L. Nevertheless, meta-analyses of randomized controlled trials suggest only small beneficial effects of vitamin D supplements on surrogate parameters of CVD risk and no reduction in CVD events. Evidence is accumulating for adverse vitamin D effects on CVD outcomes at 25OHD levels > 100 nmol/L, but the threshold may be influenced by the level of physical activity. In conclusion, dose-response relationships indicate deleterious effects on the musculoskeletal system and probably on the cardiovascular system at circulating 25OHD levels < 40–60 nmol/L and >100 nmol/L. Future studies should focus on populations with 25OHD levels < 40 nmol/L and should avoid vitamin D doses achieving 25OHD levels > 100 nmol/L.

## 1. Introduction

During the last two decades, the scientific interest in vitamin D has increased exponentially, as indicated by the fact that 65% of the 71,000 vitamin D articles available in the US National Library of Medicine by February 2017 have been published since 1997 [1]. However, the importance of vitamin D for bone health has already been known for almost 100 years. In the early 1920s, vitamin D was found to cure rickets, a bone disease that occurred endemically in infants and toddlers in many European countries and North America during the industrialization in the 19th and early 20th century [2, 3]. In some cities, up to 80% of children were afflicted by rickets [3]. Rickets prophylaxis was first performed by the administration of UV-irradiated ergosterol using doses of up to 5 mg ergosterol [4]. As early as in the 1920s, it was also recognized that administration of these doses was associated with soft tissue calcification in some children [4], indicating that beneficial vitamin D effects on bone health may lead to adverse effects on the cardiovascular system. Nowadays, rickets prophylaxis is performed with a daily dose of 400 IU vitamin D. This dose can be regarded as effective and safe [5, 6]. Although the importance and safety of vitamin D in infants are well understood, the relevance of vitamin D for the musculoskeletal and the cardiovascular system still remains a topic of scientific interest that has been extensively investigated both in experimental animals and in humans during recent years. However, the focus has moved from infancy to geriatrics, since low vitamin D status, bone diseases, and cardiovascular diseases are all prevalent in this age group [7–9].

The present narrative review gives an overview of the effects of vitamin D on the musculoskeletal and cardiovascular system. Results of experimental studies, cohort studies, Mendelian randomization studies, and randomized controlled trials (RCTs) are used to discuss both beneficial and potentially harmful vitamin D effects. Particular emphasis is paid to those studies that achieve a high level of scientific evidence such as Mendelian randomization studies and meta-analyses of RCTs. Special attention is also paid to the dose-response relationship of vitamin D with clinical outcomes.

## 2. Research Strategy

A systematic literature search in PubMed was performed without language restrictions for relevant publications released until the end of February 2017. The following search terms were used: “vitamin D” or “vitamin D supplementation” or “cholecalciferol” or “25-hydroxyvitamin D” or “VDR knockout” or “1*α*-hydroxylase deletion” or “CYP27B1 deletion” or “CYP2R1 deletion” and “bone” or “rickets” or “osteomalacia” or “osteoporosis” or “fracture” or “falls” or “cardiovascular disease” or “heart failure” or “hypertension” or “cardiovascular mortality” or “myocardial infarction” or “stroke.” Personal collections of articles on this topic as well as references from selected articles were also used to extend the search. Some articles were not cited due to space limitations.

## 3. Vitamin D Metabolism and Actions

Adequate vitamin D supply can be achieved through dietary vitamin D intake, vitamin D supplement use, and/or skin exposure to solar ultraviolet (UV) B radiation. In the absence of skin synthesis of vitamin D, a daily oral dose of 400 IU and 800 IU is regarded to be adequate for infants and the general population beyond infancy, respectively [10]. The upper tolerable intake level is age dependently considered to be 1000 to 4000 IU [6, 11]. For adult patients who are at risk of inadequate vitamin D status, the Endocrine Society recommends a daily vitamin D dose of 1500 to 2000 IU and considers daily doses of up to 10,000 IU as safe (Table 1).

**Figure 1:**
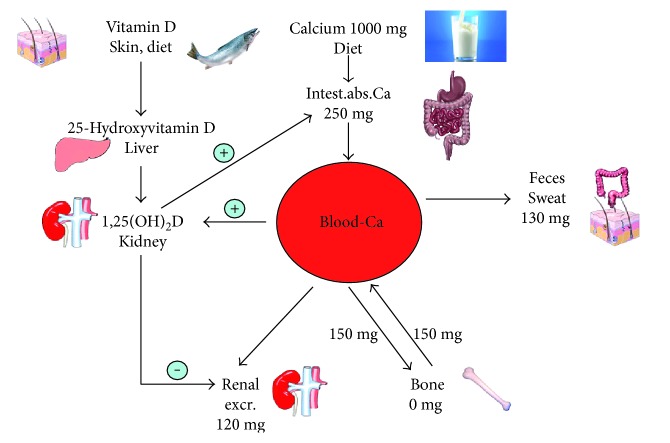
Calcium and vitamin D metabolism at the example of a young adult.

Vitamin D is activated by a hepatic 25-hydroxylation (principle hydroxylase: CYP2R1-hydroxylase; additional hydroxylase: CYP27R1-hydroxylase) and a renal a 1*α*-hydroxylation (CYP27B1-hxdroxylase) into its active hormonal form 1,25-dihydroxyvitamin D (1,25[OH]_2_D) (Figure 1). The best indicator for defining human vitamin D status is the circulating 25-hydroxyvitamin D (25OHD) concentration. The usefulness of this parameter for assessing vitamin D-dependent biochemical actions can be explained by the fact that various local tissues including enterocytes also possess 1*α*-hydroxylase activity [12] and that 1,25(OH)_2_D is reduced in case of deficient 25OHD levels [13]. However, classifications of circulating 25OHD concentrations are inconsistent: the North American Institute of Medicine (IOM) [6] has classified values < 30 nmol/L as deficient, 30–49.99 nmol/L as insufficient, 50–125 nmol/L as adequate, and >125 nmol/L as potentially harmful. The Endocrine Society considers 25OHD levels < 50 nmol/L as deficient and levels between 50 and 74.99 nmol/L as insufficient [14]. Moreover, from their clinical practise guideline [14], it can indirectly be assumed that the Endocrine Society considers 25OHD levels of 75 up to 250 nmol/L as adequate and >250 nmol/L as potentially harmful.

Vitamin D plays a pivotal role in the regulation of calcium and phosphate metabolism and the maintenance of adequate blood levels of these minerals. In case of low serum ionized calcium concentrations (e.g., during low dietary calcium intake), renal 1,25(OH)_2_D synthesis is activated by parathyroid hormone (PTH), whereas PTH and renal 1*α*-hydroxylation of 25OHD are suppressed by high plasma calcium levels [15]. Renal 1*α*-hydroxylase is also suppressed by fibroblast growth factor- (FGF-) 23, a phosphaturic hormone which is secreted by bone cells. FGF-23 is stimulated by high serum phosphate levels and promotes phosphaturia to maintain serum phosphate levels within the normal range [15].

With respect to beneficial and potential harmful vitamin D effects, it is noteworthy that no evidence of a threshold in calcium absorption rate was found with a serum 25OHD level ranging from deficient concentrations up to 150 nmol/L [16–18]. Since both an increase in oral calcium intake and higher serum 25OHD levels are associated with a rise of intestinal absorbed calcium, calcium and vitamin D can replace each other relative to their effects on calcium supply (Figure 1). In line with this assumption, circulating 25OHD levels > 45 nmol/L can ensure low PTH levels even when the calcium intake level is less than 800 mg/day, while a calcium intake above 1200 mg/day is not sufficient to maintain adequate serum PTH, as long as vitamin D status is below 45 nmol/L [19]. Highest PTH levels have been reported in individuals with 25OHD levels < 25 nmol/L and calcium intakes < 800 mg/day [19].

Besides its role in maintaining mineral homeostasis, 1,25(OH)_2_D has been shown to play an important role in the musculoskeletal and the cardiovascular system. Briefly, in skeletal muscle cells, vitamin D affects cell proliferation and differentiation and the transport of calcium and phosphate across skeletal muscle cell membranes, suppresses the expression of myostatin, a negative regulator of muscle mass, upregulates the expression of follistatin and insulin-like growth factor 2, induces the expression of a number of myogenic transcription factors, regulates muscle cell differentiation by inducing cell cycle arrest, prevents muscular degeneration, and reverses myalgia [20]. In the cardiovascular system, vitamin D downregulates proinflammatory cytokines, metalloproteinases, and natriuretic peptides [21, 22] and upregulates matrix gla protein, anti-inflammatory cytokines, and inhibitors of metalloproteinases [22]. It is however also noteworthy that calcium supplements increase the risk of CVD events, especially myocardial infarction [23].

Hypercalcemia is the hallmark of vitamin D intoxication. Hypercalcemia promotes vascular calcification by the transition of contractile vascular smooth muscle cells into the osteoblast-like phenotype [24]. It has been stated that vitamin D intoxication is observed when circulating 25OHD levels are greater than 374 nmol/L [25]. However, with respect to the risk of hypercalcemia, others have selected 220 nmol/L as a healthy adult NOAEL (no observed adverse effect level) for circulating 25OHD [26]. Despite the aforementioned threshold levels, it is noteworthy that long-term results of vitamin D on plasma calcium are very limited and, according to the IOM, there continues to be large uncertainty about the progressive health effects for regular ingestion of even moderately high amounts of vitamin D in the long run [26].

## 4. Vitamin D Deficiency, Bone Disorders, and Cardiovascular Diseases

The consequences of vitamin D deficiency on the human musculoskeletal system have long been known and also well characterized in experimental animals. Therefore, experimental data on vitamin D deficiency and the skeleton during recent years were most of all confirmative, whereas experimental data on the cardiovascular system have provided important new insights on potential interaction between vitamin D and the cardiovascular system. Findings in experimental animals, infants, and adults are summarized below.

### 4.1. Experimental Data

Mice lacking the vitamin D receptor (VDR) develop hypocalcemia, severe hyperparathyroidism, elevated plasma levels of alkaline phosphatase, and the typical features of rickets. Normalization of impaired mineral homeostasis in VDR knockout mice fed a diet supplemented with high concentrations of calcium (2%) and phosphorus (1.25%) is reported to reverse the malformation of the bone and the growth retardation as well [27], indicating that the most important action of the VDR in skeletal growth, maturation, and remodeling is its role in intestinal calcium absorption [28]. As expected, targeted ablation of the CYP27B1 gene (1*α*-hydroxylase gene) in mice results in hypocalcemia, secondary hyperparathyroidism, retarded growth, and the skeletal abnormalities characteristic of rickets as well [29]. In CYP2R1 knockout mice, circulating 25OHD is reduced by more than 50% and it has been suggested that in some patients with rickets CYP2R1 mutations may be responsible for the disease [30].

In the cardiovascular system, VDR deletion results in elevated production of renin and angiotensin II, leading to hypertension and cardiac hypertrophy [31, 32]. Treatment of VDR knockout mice with the ACE inhibitor captopril reduces cardiac hypertrophy and normalizes atrial natriuretic peptide expression [33]. Cardiomyocyte-specific deletion of the VDR also results in cardiac hypertrophy, and treatment of neonatal cardiomyocytes with 1,25(OH)_2_D is partially able to suppress hypertrophy [31]. Moreover, vitamin D deficiency stimulates renin expression in normal mice, whereas injection of 1,25(OH)_2_D reduces renin synthesis [31]. This protective role of 1,25(OH)_2_D on the cardiovascular system seems to be independent of plasma calcium and phosphate levels [34]. Deletion of the VDR as well as diets low in vitamin D content also stimulates osteoblast-like cell formation of vascular smooth muscle cells and aortic calcification [35].

### 4.2. Infancy

Rickets is the principal vitamin D-deficiency disease in infants. In the majority of studies in which circulating 25OHD has been measured in toddlers with rickets living in Europe, concentrations were <12.5 nmol/L [36]. However, higher 25OHD levels have also been reported [36] and dietary calcium deprivation rather than vitamin D deficiency may have been the cause of rickets in these cases. Some RCTs in children with 25OHD levels > 25 nmol/L but <50 nmol/L have demonstrated that the best therapeutic response is seen with a combination of calcium with vitamin D and if 25OHD levels achieve values > 40 to 50 nmol/L [37–39]. Collectively, data in infants support experimental and biochemical findings of jointed vitamin D and calcium effects on bone health. The risk of rickets progressively increases at circulating 25OHD < 40 nmol/L.

Some infants with rickets also develop cardiac problems: In a series of 61 cases of infants with rickets and heart failure [40], almost all patients had low circulating levels of 25OHD (mean values: 18.5 nmol/L), low plasma calcium concentrations, and low plasma phosphate concentrations, whereas PTH levels were markedly elevated. The vast majority of infants responded to treatment with calcium, vitamin D, and cardiotonics, indicating that vitamin D (and calcium) may have played an important role in the pathogenesis of the cardiac problems. The results are supported by an RCT in 80 infants with heart failure [41] and mean 25OHD levels of 35 nmol/L, in which treatment with 1000 IU vitamin D daily suppressed PTH levels, the proinflammatory cytokines interleukin-6 and tumor necrosis factor-*α*, and increased the anti-inflammatory cytokine interleukin-10 as well as left ventricular ejection fraction significantly.

### 4.3. Adulthood

It is well known that in adults prolonged and severe vitamin D deficiency (<12.5 nmol/L) can cause osteomalacia, a musculoskeletal disorder that is associated with diffuse joint and bone pain, muscle weakness, difficulty in walking, bone demineralization, and increased fracture risk. There is also evidence that 25OHD levels already below 25 nmol/L lead to osteomalacia in the long run [42]. Similar to rickets, osteomalacia is associated with hypocalcemia, hypophosphatemia, and severe hyperparathyroidism. Osteomalacia has been reported to be common in elderly women in the UK [43]. In Turkish immigrants in Germany, a high prevalence of vitamin D deficiency (78% < 50 nmol/L), secondary hyperparathyroidism (40% of those with low 25OHD levels), and generalized bone pain has also been reported, especially in veiled women [44]. Earlier data indicate that subclinical osteomalacia can already be corrected by relatively low doses of alfacalcidol (0.5 micrograms daily) or plain vitamin D (1000 IU daily) given for three months [45]. Moreover, 400 IU of vitamin D with 600 mg calcium daily was already adequate to increase bone mineral density significantly in low-income Bangladeshi women with low outdoor activities [46]. A histomorphometric analysis of iliac crest bone biopsies and circulating 25OHD in 675 patients demonstrated that pathologic bone mineralization was most prevalent in patients with 25OHD levels < 25 nmol/L [47]. The threshold for the absence of mineralization defects was 75 nmol/L. The investigators therefore concluded that together with a sufficient calcium intake, circulating 25OHD levels > 75 nmol/L should be ensured to maintain skeletal health. It is however noteworthy that the aforementioned investigation was an observational study and therefore cannot prove causality. Caution is necessary in recommending 75 nmol/L because a daily vitamin D supplement of 3800 to 5000 IU would be necessary to guarantee circulating 25OHD level of 75 nmol/L in almost all adults [48]. These doses would reach or exceed the UL and would be clearly above the IOM recommendation for older adults (Table 1).

Since the muscle is a target tissue for vitamin D, vitamin D deficiency is also discussed to contribute to an increased risk of falls (and fractures) in the elderly. Numerous meta-analyses of RCTs have summarized the results of vitamin D on the risk of falls/falling. Findings support the assumption that in the elderly the risk of falls/falling is influenced by baseline 25OHD levels, achieved 25OHD level, and calcium coadministration. Briefly, in a meta-analysis of 26 RCTs that enrolled 45,782 participants [49], vitamin D use was also associated with statistically significant reduction in the risk of falls (odds ratio 0.86 [(95% CI: 0.77–0.96]). This effect was more prominent in patients who were vitamin D deficient at baseline and in studies in which calcium was coadministered with vitamin D. In community dwellers [50], vitamin D did not reduce the rate of falls or risk of falling. However, it was concluded that it may do so in people with lower 25OHD levels before treatment. In patients of nursing care facilities, a group that is known to have a high prevalence of vitamin D deficiency, vitamin D supplementation reduced the rate of falls to 0.72 (95% CI, 0.55 to 0.95) [51]. In another meta-analysis of 8 RCTs [52], based on 2426 individuals, supplemental vitamin D in a dose of 700–1000 IU a day reduced the risk of falling among older individuals by 19% and to a similar degree as active forms of vitamin D. It was concluded from this meta-analysis that doses of supplemental vitamin D of less than 700 IU may not reduce the risk of falling among older individuals and that circulating 25OHD levels of 60 nmol/L should be achieved.

Data of RCTs on vitamin D and fracture risk support results on vitamin D and falls: the combined vitamin D and calcium administration was able to reduce fracture risk significantly only in institutionalized elderly individuals but not in community dwellers [53], probably because of lower baseline 25OHD levels in the former group of individuals. In pooled participant level data of RCTs, a 30% reduction in the risk of hip fracture and a 14% reduction in the risk of any nonvertebral fracture were shown if on the basis of actual intakes daily vitamin D intakes were at least 800 IU [54]. In line with the findings of the aforementioned meta-analysis on falls [52], a dose-response relationship was suggested with the highest and lowest fracture risk at 25OHD levels < 30 nmol/L and >61 nmol/L, respectively. However, it is noteworthy that the dose-response relationships on falls and fractures investigated by Bischoff-Ferrari et al. [52, 54] were only exploratory analyses of RCTs and can thus be subject to unexplained bias.

In total, results in infants and adults indicate a dose-response relationship between circulating 25OHD and the musculoskeletal system with the highest risk below 25 nmol/L and a low risk if a level of approximately 40 to 60 nmol/L is achieved.

Regarding vitamin D and CVD, it is noteworthy that data from RCTs on “hard” clinical endpoints are scarce. Therefore, epidemiological data have to be taken into account as well. In a meta-analysis of prospective cohort studies based on more than 20,000 individuals [55], adjusted risk of cardiovascular mortality was 57% higher in the lowest 25OHD category than in the highest 25OHD category. The Whitehall study [56], a large prospective cohort study of older men living in the UK, indicates that higher concentrations of 25OHD are inversely and approximately linearly (log-log scale) associated with age- and season-adjusted vascular mortality throughout the range of 40–90 nmol/L. After additional adjustment for prior disease and cardiovascular risk factors, a doubling in 25OHD concentration was associated with 20% [95% CI: 9–30%] lower vascular mortality. In a milestone publication of a European consortium of eight prospective studies [57], including seven general population cohorts, individual patient data and standardized 25OHD data were used to assess the association of 25OHD with all-cause and cause-specific mortality. Compared to participants with adequate 25OHD concentrations (75 to 99.99 nmol/L), the adjusted hazard ratios (with 95% Cl) for CVD mortality in the 25OHD groups with 40 to 49.99, 30 to 39.99, and <30 nmol/L were 1.65 (1.1.39–1.97), 1.61 (1.46–1.77), and 2.21 (1.50–3.26), respectively. In line with these findings, a 2017 meta-analysis of 34 cohort studies with more than 180,000 participants [58] reported a progressive increase of total CVD events at circulating 25OHD levels < 50 nmol/L but no association of 25OHD with CVD events at levels between 50 and 137 nmol/L. With respect to CVD mortality, the risk increased constantly at circulating 25OHD levels < 100 nmol/L [58].

Despite these promising epidemiological data regarding an effect of vitamin D status on CVD outcome, cohort studies are subject to residual confounding. Therefore, a Danish approach using an observational study design together with a Mendelian randomization analysis [59] is vitally important. Mendelian randomization takes advantage of lifelong differences in vitamin D status attributable to genetic variants and is hence not confounded by lifestyle factors. In the Danish investigation, the odds ratio for an observational multivariable-adjusted 20 nmol/L lower 25OHD concentration was 1.13 (95% CI: 1.03 to 1.24) for cardiovascular mortality but was 0.77 (95% CI: 0.55 to 1.08) for a genetically determined 20 nmol/L lower 25OHD level. Similarly, the observational multivariable-adjusted hazard ratios for a 25 nmol/L decrease in 25OHD were significantly higher for ischemic heart disease and myocardial infarction, whereas the hazard ratios for a genetically 25 nmol/L decrease were not [60]. Results are an indication that no premature conclusions should be drawn solely based on observational data.

Several RCTs have investigated surrogate parameters of cardiovascular risk such as blood pressure and arterial stiffness. Regarding blood pressure, a meta-analysis incorporating individual patient data of 46 RCTs came to the conclusion that vitamin D supplementation is ineffective as an agent for lowering blood pressure [61]. In RCTs with initial 25OHD levels > 40 nmol/L, even a clear increase in 25OHD levels did not influence systolic or diastolic blood pressure [62, 63]. However, in a Mendelian randomization approach including up to 108,173 individuals from 35 studies [64], each 10% increase in genetically determined 25OHD concentration was associated with a significant change of −0·29 mm Hg in diastolic blood pressure, a significant change of −0·37 mm Hg in systolic blood pressure, and an 81% decreased odds of hypertension, indicating that in the long run vitamin D might have a small but significant beneficial effect on blood pressure. A meta-analysis of RCTs on arterial stiffness [65] reported nonsignificant reductions in pulse wave velocity (standardized mean difference = −0·10; 95% CI: −0·24, 0·04) and augmentation index (−0·15; 95% CI: −0·32, 0·02), the latter being a measure of the enhancement of central aortic pressure, by vitamin D supplementation in the range of 1000 to 5700 IU/day. Out of the included 18 studies, 11 had mean 25OHD levels < 50 nmol/L, 4 between 50 and 75 nmol/L, and 2 > 75 nmol/L at recruitment, whereas one study provided no 25OHD data.

Regarding CVD events, a meta-analysis of RCTs could not demonstrate a beneficial vitamin D effect on myocardial infarction or stroke [66] and these results were also confirmed by another more recent meta-analysis [67]. However, this recent meta-analysis [67] reported a 17% reduction in heart failure events by vitamin D supplementation. Nevertheless, it is noteworthy that results were largely influenced by a secondary analysis of only one large trial. In a very recent large RCT in elderly patients with initial 25OHD levels of 63.7 nmol/L [68], monthly high-dose vitamin D supplementation did not prevent CVD events. Moreover, a systematic Cochrane review on vitamin D supplementation for prevention of mortality in adults [69] showed no beneficial effect on CVD mortality.

Collectively, surrogate parameters of cardiovascular risk do not exclude the possibility of small beneficial vitamin D effects on CVD risk. However, the dose-response relationship is yet poorly understood and there is currently no convincing evidence that potential beneficial vitamin D effects on the cardiovascular system lead to a reduction of CVD events. More RCTs in individuals with deficient 25OHD levels (i.e., <30 nmol/L) are needed.

## 5. Harmful Vitamin D Effects on the Musculoskeletal and Cardiovascular System

### 5.1. Vitamin D and the Musculoskeletal System

Although calcium release from the bone is considered to be the most important cause of hypercalcemia seen in vitamin D intoxication [70], adverse effects of toxic vitamin D doses on the musculoskeletal system are almost completely lacking in experimental animals or infants. In adults, however, some recent investigations have reported adverse effects on the musculoskeletal system at higher circulating 25OHD levels or at higher vitamin D doses: a population-based prospective study in older men [71] reported a U-shaped association of circulating 25OHD levels with fracture risk, with the highest risk for patients not only in the lowest 25OHD quantile (≤36 nmol/L) but also in the highest quantile (>72 to ≤148 nmol/L) (reference group: >59 to ≤72 nmol/L). Results are confirmed by a large placebo-controlled trial in 2256 community-dwelling women, aged 70 years or older [72]. Compared with women in the placebo group, bolus administration of vitamin D (500,000 IU vitamin D3 once a year, equivalent to 1370 IU/daily, for 3 years) resulted in a higher rate of falls (83.4 versus 72.7 per 100 person-years, *P* = 0.03) and a higher rate of fractures (4.9 versus 3.9 per 100 person-years, *P* = 0.047). The increased likelihood of falls and fractures in the vitamin D group was exacerbated in the 3-month period immediately following the annual dose. Levels of 25OHD increased in the vitamin D group at 1 month after dosing to approximately 120 nmol/L and to approximately 90 nmol/L at 3 months. Another study also reported an increase in fracture associated with vitamin D treatment [73]. Participants (4354 men, 5086 women) 75 years or older received an annual injection of 300,000 IU vitamin D2 (equivalent to 820 IU/daily) or placebo. In men, treatment had no effect on fractures. However, women treated with vitamin D had a 21% higher risk of nonvertebral fractures, an 80% higher risk of hip/femur fractures, and a 59% higher risk of hip/femur/wrist/forearm fractures. Two recent RCTs could confirm the higher risk of falls by bolus administration of vitamin D. In a cohort of 200 community-dwelling men and women, 70 years and older [74], the incidence of falls was higher in the group receiving 60,000 IU vitamin D monthly (equivalent to 2000 IU vitamin D daily) and in the group receiving 24,000 IU vitamin D plus 300 *μ*g calcifediol monthly (equivalent to 800 IU vitamin D plus 10 *μ*g calcifediol daily) than in the group receiving 24,000 IU vitamin D monthly (equivalent to 800 IU vitamin D daily) (incidence 66.9%, 66.1%, and 47.9%, resp.; *P* = 0.048). In addition, the total mean number of falls tended to be higher in the two former groups than in the latter group. Seniors reaching the highest quartile of 25OHD level at the 12-month follow-up (112–247 nmol/L) had a 5.5-fold higher odds of falling compared with those reaching the lowest quartile of 25OHD (53.2 to 75.6 nmol/L). In another study in 107 long-term care residents aged 60 and older [75], falls were more common in a high-dose vitamin D group receiving a monthly supplement of 100,000 IU vitamin D3 (equivalent to 3333 IU daily) versus a standard-dose vitamin D group receiving 400 to 1000 IU daily (1.47 versus 0.63 per person-years; *P* < .001). Fractures were uncommon and similar in both groups. Mean circulating 25OHD levels during the trial were 80 nmol/L in the high-dose group and 63 nmol/L in the standard-dose group. In total, vitamin D effects on the musculoskeletal system seem to follow a U-shaped association, with deleterious effects at low circulating 25OHD concentrations (i.e., <50 nmol/L) and also at high 25OHD concentrations. Especially, individuals achieving 25OHD levels > 100 nmol/L seem to be at an increased risk.

### 5.2. Vitamin D and the Cardiovascular System

Numerous historical and recent studies have demonstrated that supraphysiological doses of vitamin D result in vascular calcification in experimental animals and these results have already been summarized elsewhere [4, 76, 77]. Moreover, harmful cardiovascular effects of toxic vitamin D doses (resulting in 25OHD > 374 nmol/L) are well established in infants and adults [4, 6, 77, 78], but the question arises whether levels already between 100 nmol/L and 374 nmol/L are also associated with an increased CVD risk [79].

In the aforementioned milestone cohort study of a European consortium [57], 25OHD levels > 100 nmol/L were not associated with an increased risk of CVD mortality. However, the majority of samples exceeding the threshold of 100 nmol/L originated from a German cohort of apparently healthy, middle-aged individuals and may thus not be representative for individuals in the clinical setting. In two huge Israeli and Danish data analyses in patients from the general practise sector [80, 81], an inverse J-shaped association of circulating 25OHD with CVD morbidity and mortality was reported. Morbidity and mortality were lowest at 25OHD levels between 50 and 90 nmol/L and increased again above this range. In another prospective cohort study in cardiac surgical patients [82], a U-shaped association between circulating 25OHD and the risk of major adverse cardiac and cerebrovascular events has been reported. Risk was highest at both circulating 25OHD levels < 30 nmol/L and >100 nmol/L. A recent RCT in advanced heart failure provided further evidence for adverse vitamin D effects in CVD patients [83]. A daily vitamin D supplement of 4000 IU for 3 years resulted in a greater need for mechanical circulatory support implants, especially in patients with initial circulating 25OHD concentrations ≥ 30 nmol/L. They also achieved median in-study 25OHD levels > 100 nmol/L. The underlying mechanism for this effect remains unclear at present but may be related to elevated plasma calcium levels. In this study, the incidence of hypercalcemia (plasma calcium > 2.75 mmol/L) was in the vitamin D and placebo group (6.2% and 3.1%, resp., *P* = .192). Generally, it seems that oral vitamin D doses resulting in mean circulating 25OHD levels of 75 to 160 nmol/L do not lead to hypercalcemia [79]. However, in the aforementioned RCT, vitamin D administration resulted in a significant increase in plasma calcium, although mean calcium levels remained within the reference range [82]. A similar effect has already been reported in an earlier RCT in heart failure [84]. Importantly, the ARIC (Atherosclerosis Risk in Communities) study reported that high plasma calcium was independently associated with greater risk of incident heart failure [85]. Heart failure incidence was lowest at calcium levels of 2.25 mmol/L and increased progressively up to 2.75 mmol/L [85]. Moreover, a meta-analysis of observational data indicates a statistically positive association between plasma calcium and CVD [86].

## 6. Interactions of Vitamin D with the Musculoskeletal and Cardiovascular System

An increase in plasma calcium does not only result from excessive vitamin D doses but can also be due to other reasons. Briefly, hypokinesia and immobilization are associated with a significant increase in plasma calcium and phosphate and a decrease in circulating 1,25(OH)_2_D levels [87, 88]. Similarly, postmenopausal bone loss is associated with a significant rise in plasma calcium [89]. According to the Utah paradigm of bone biology [90], the increase in plasma calcium and decrease in circulating 1,25(OH)_2_D in postmenopausal women and individuals with sedentary lifestyle can be explained by a loss of bone mass due to estrogen deficiency or muscle loss, subsequently leading to an influx of calcium into soft tissues such as vessels and kidneys.

Vascular calcification has been identified as a risk factor for CVD mortality [91] and a predictor of poorer 5-year survival [92]. The inverse relationship between the amount of vascular and skeletal calcium can explain why vascular calcification is often associated with osteoporosis [93, 94]. While vitamin D supplementation appears logical in case of inadequate vitamin D supply to increase the amount of intestinally absorbed calcium and thus to prevent musculoskeletal diseases, such a measure appears questionable when plasma calcium levels are already elevated due to immobilization-induced or estrogen deficiency-induced calcium release from the bone. Therefore, scepticism is necessary regarding an American Geriatrics Society consensus statement [95]. They recommend up to 4000 IU daily of vitamin D supplementation for prevention of falls in older adults. The effect of moderately high daily vitamin D doses on the cardiovascular system is far from clear. Since this amount may further increase plasma calcium levels (see before), caution is needed in administering vitamin D doses of 4000 IU in the clinical setting.

It is however intriguing that physical activity and remobilization have hypocalcemic effects [96] and are associated with an increase in circulating 1,25(OH)_2_D [87, 88, 97]. Although physically active individuals have higher 25OHD levels than individuals with sedentary lifestyle, indicating an increase in intestinal calcium absorption, the surplus of absorbed calcium is usually excreted via sweat or deposed in the skeleton [98]. This effect of physical activity on calcium metabolism is thus in line with findings that traditionally living individuals with abundant UVB exposure have a lifelong low CVD risk [99], although circulating 25OHD levels in these groups clearly exceed 100 nmol/L [100]. However, the high circulating 25OHD levels in these groups cannot a priori be considered as safe for an aging westernized society with sedentary lifestyle. Likely, results on circulating 25OHD and CVD outcomes obtained in Mendelian randomization studies in patients with a high CVD risk should not be extrapolated to young healthy individuals.  Table 2 presents a potential dose-response relationship of circulating 25OHD with musculoskeletal and cardiovascular outcomes.

## 7. Conclusions

There is accumulating evidence that circulating 25OHD levels < 40–60 nmol/L are nonlinearly related to an increased risk of musculoskeletal diseases and probably also to an increased CVD risk. The classification of the North American IOM [6] and of several European Nutrition Societies [10] of circulating 25OHD levels > 50 nmol/L as adequate is in line with these findings. Recent results demonstrate that a daily vitamin D supplement of 800 IU is able to achieve circulating 25OHD levels in almost all young female adults in winter [101]. Elderly people usually require on average a daily dose of ≤400 IU to achieve 25OHD levels > 50 nmol/L [102]. These data concur with official recommendations of an oral intake of 800 IU vitamin D daily beyond infancy in the absence of skin synthesis of vitamin D [10].

The threshold of harmful vitamin D effects is probably influenced by the level of physical activity. In the clinical setting, caution is needed in administering vitamin D doses resulting in circulating 25OHD levels > 100 nmol/L. Some statements, such as a daily vitamin D intake of up to 4000 IU for the prevention of falls [95] or that a daily intake of up to 10,000 IU vitamin D is safe [14], should therefore be reconsidered. In the future, RCTs with multiple outcomes and multivariate meta-analyses of RCTs are needed to assess the health effects of vitamin D supplements on the musculoskeletal and cardiovascular system.

## Figures and Tables

**Table 1:** Daily vitamin D recommendations and daily upper tolerable intake levels by different organizations [6, 10–12].

Life Stage Group	Recommendations			Upper tolerable intake level		
	D-A-CH^1,2^	IOM^3^	ES^4,5^	EFSA^6^	IOM	ES
Infants						
0–6 months	400	400	400–1000	1000	1000	2000
6 to 12 months	400	400	400–1000	1000	1500	2000
Children						
1–3 yr	800	600	600–1000	2000	2500	4000
4–8 yr	800	600	600–1000	2000	3000	4000
Males						
9–13 yr	800	600	600–1000	2000–4000	4000	4000
14–18 yr	800	600	600–1000	4000	4000	4000
19–30 yr	800	600	1500–2000	4000	4000	10,000
31–50 yr	800	600	1500–2000	4000	4000	10,000
51–70 yr	800	600	1500–2000	4000	4000	10,000
70+ yr	800	800	1500–2000	4000	4000	10,000
Females						
9.13 yr	800	600	600–1000	4000	4000	4000
14–18 yr	800	600	600–1000	4000	4000	4000
19–30 yr	800	600	1500–2000	4000	4000	10,000
31–50 yr	800	600	1500–2000	4000	4000	10,000
51–70 yr	800	600	1500–2000	4000	4000	10,000
70+ yr	800	800	1500–2000	4000	4000	10,000
Pregnancy						
14–18 yr	800	600	600–1000	4000	4000	4000
19–30 yr	800	600	1500–2000	4000	4000	10,000
31–50 yr	800	600	1500–2000	4000	4000	10,000
Lactation						
14–18 yr	800	600	600–1000	4000	4000	4000
19–30 yr	800	600	1500–2000	4000	4000	10,000
31–50 yr	800	600	1500–2000	4000	4000	10,000

^1^German, Austrian, Swiss Nutrition Societies. ^2^In the absence of skin synthesis of vitamin D. ^3^Institute of Medicine. ^4^Endocrine Society. ^5^For patients at risk for 25-hydroxyvitamin D levels < 50 nmol/L. ^6^European Food Safety Authority. Vitamin D data are presented as international units.

**Table 2:** Suggested dose-response relationship of circulating 25-hydroxyvitamin D with musculoskeletal and cardiovascular disease.

25-Hydroxyvitamin D concentration	Musculoskeletal system	Cardiovascular system
<12.5 nmol/L	Rickets ↑↑, osteomalacia ↑↑ Elderly people: falls ↑↑, fractures ↑↑	CVD events ↑ (?)
12.5–24.99 nmol/L	Rickets ↑, osteomalacia ↑ Elderly people: falls ↑↑, fractures ↑↑	CVD events ↑ (?)
25.0–49.99 nmol/L	Elderly people: falls ↑, fractures ↑	CVD surrogate parameters probably adversely affected
50.0–100.0 nmol/L	Adequate muscle and bone function	Adequate cardiovascular function
>100 nmol/L	Elderly people: falls ↑, fractures ↑	CVD events ↑ (?)

CVD: cardiovascular disease events; (?): probably; ↑: elevated; ↑↑: markedly elevated.
